# Wnt4 Enhances Murine Hematopoietic Progenitor Cell Expansion Through a Planar Cell Polarity-Like Pathway

**DOI:** 10.1371/journal.pone.0019279

**Published:** 2011-04-26

**Authors:** Krista M. Heinonen, Juan Ruiz Vanegas, Deborah Lew, Jana Krosl, Claude Perreault

**Affiliations:** 1 Institute for Research in Immunology and Cancer, Université de Montréal, Montreal, Quebec, Canada; 2 Department of Medicine, Université de Montréal, Montreal, Quebec, Canada; 3 Division of Hematology, Maisonneuve-Rosemont Hospital, Montreal, Quebec, Canada; Centro Cardiologico Monzino, Italy

## Abstract

**Background:**

While the role of canonical (β-catenin-mediated) Wnt signaling in hematolymphopoiesis has been studied extensively, little is known of the potential importance of non-canonical Wnt signals in hematopoietic cells. Wnt4 is one of the Wnt proteins that can elicit non-canonical pathways. We have previously shown that retroviral overexpression of Wnt4 by hematopoietic cells increased thymic cellularity as well as the frequency of early thymic progenitors and bone marrow hematopoietic progenitor cells (HPCs). However, the molecular pathways responsible for its effect in HPCs are not known.

**Methodology/Principal Findings:**

Here we report that Wnt4 stimulation resulted in the activation of the small GTPase Rac1 as well as Jnk kinases in an HPC cell line. Jnk activity was necessary, while β-catenin was dispensable, for the Wnt4-mediated expansion of primary fetal liver HPCs in culture. Furthermore, Jnk2-deficient and Wnt4 hemizygous mice presented lower numbers of HPCs in their bone marrow, and Jnk2-deficient HPCs showed increased rates of apoptosis. Wnt4 also improved HPC activity in a competitive reconstitution model in a cell-autonomous, Jnk2-dependent manner. Lastly, we identified Fz6 as a receptor for Wnt4 in immature HPCs and showed that the absence of Wnt4 led to a decreased expression of four polarity complex genes.

**Conclusions/Significance:**

Our results establish a functional role for non-canonical Wnt signaling in hematopoiesis through a pathway involving Wnt4, Fz6, Rac1 and Jnk kinases.

## Introduction

Wnt signaling proteins are highly conserved and essential for organismal patterning from nematodes to man [1]–[3]. Both Wnt ligands and their receptors, Frizzleds (Fz), form multigene families, with a large number of possible ligand-receptor interactions. Moreover, the variety of different intracellular events potentially induced by the Wnt/receptor interactions further adds to the complexity of Wnt signaling. Traditionally, intracellular Wnt signaling pathways have been divided in two broad categories: 1) the canonical pathway, dependent on the stabilization of β-catenin and its translocation to the nucleus, and 2) the non-canonical pathways that comprise all β-catenin-independent Wnt-induced signaling events [1]–[3]. The intracellular molecules involved in non-canonical Wnt signaling range from Rho and Rac GTPases and Jnk kinases to mediators of intracellular calcium fluxes, Src family kinases and Nlk [4]. Non-canonical Wnt signaling has pleiotropic effects on cell polarity, directed motility, morphogenesis and was shown to regulate mammalian stem cell biology in at least two situations: Wnt7a drives the symmetric expansion of skeletal muscle satellite stem cells while Wnt11 orchestrates specification of human embryonic stem cells toward hematopoietic lineage [5], [6].

The role of the canonical Wnt pathway in vertebrate hematopoiesis has been studied extensively and has generated some controversy [7], [8]. Initial studies implicating Wnt signaling in hematopoietic stem cell (HSC) biology have been challenged by a series of reports, indicating that β-catenin was dispensable for normal adult hematopoiesis, and that its forced stabilization resulted in loss of HSC activity through exhaustion (reviewed in [8]). More recently, three groups have provided further proof in favor for Wnts in HSC biology: 1) deletion of β-catenin in hematopoietic cells during development resulted in impaired HSC self-renewal during serial transplants [9]; 2) lack of Wnt3a in the fetal liver induced a severe, cell-autonomous HSC self-renewal defect [10]; and 3) inhibition of Wnt signaling in the osteoblastic HSC niche in the bone marrow (BM) irreversibly decreased the capacity of the HSC to reconstitute a secondary host [11]; In the latter three studies, preservation of HSC self-renewal was attributed specifically to the canonical Wnt pathway. Therefore, the consensus that can be drawn from the current literature is that canonical Wnt signaling plays a role in HSCs and the dosage of β-catenin is of major importance in determining the outcome of a canonical Wnt signal.

Little is known about the role of non-canonical Wnt signaling in cells committed to the hematopoietic lineage [12]. Progress in our understanding of non-canonical Wnt signaling is complicated by the fact that no single strategy allows global (yet specific) inhibition or monitoring of the heterogeneous non-canonical Wnt pathways. Therefore, evaluation of the role of non-canonical Wnt pathways hinges on the analysis of the signals elicited by specific Wnt proteins in discrete cell populations. Wnt5a has been shown to activate non-canonical signaling in Lin^-^Sca1^+^cKit^hi^ (LSK) hematopoietic progenitors, which include HSCs, and to improve HSC maintenance and function [13]–[15]. However, it is not known what non-canonical signaling pathways are used by Wnt5a in HSCs nor is it clear by what mechanisms Wnt5a regulates HSC repopulation [12]. We have previously reported that hematopoietic overexpression of Wnt4 resulted in increased thymic cellularity and augmented the size of the LSK pool in the BM of irradiated recipient mice [16], in particular that of lymphoid-primed multipotent progenitors (LMPP), which express high levels of Flt3 and have been shown to participate in thymus seeding [17]–[19]. Wnt4 is a prototypical non-canonical Wnt: in a proper context it can trigger β-catenin-independent signals [20], [21]. Nevertheless, events downstream of Wnt4 induction cannot be automatically attributed to non-canonical signaling because, like all non-canonical Wnts, Wnt4 can also elicit β-catenin signaling in a receptor-dependent fashion [22]. Here we explore further the pre-thymic effects of Wnt4 on hematopoietic progenitor cells (HPC) and demonstrate that Wnt4 activates Rac1 and Jnk in HPCs, and that both Jnk activity and the presence of the receptor Fz6 are necessary for Wnt4-mediated LSK expansion. We also show that Wnt4, Jnk2 and Fz6 are all involved in BM LSK maintenance at steady state. Lastly, Wnt4 improves the competitive reconstitution capacity of LSKs in a manner that is at least partially dependent on Jnk kinases. Our results show that Wnt4 improves HPC function in a Jnk- and Fz6-dependent manner.

## Results

### Wnt4 activates JNK kinase in hematopoietic progenitor cells

The most studied Wnt signaling pathway in hematopoietic cells remains the β-catenin-dependent canonical pathway. However, there are several non-canonical pathways that have been described in other cell types, and the signaling pathway activated by a given Wnt is to a large extent cell type dependent. Therefore, we wanted to determine which signaling pathways were biologically relevant in the context of Wnt4 in immature hematopoietic cells.

**Figure 1:**
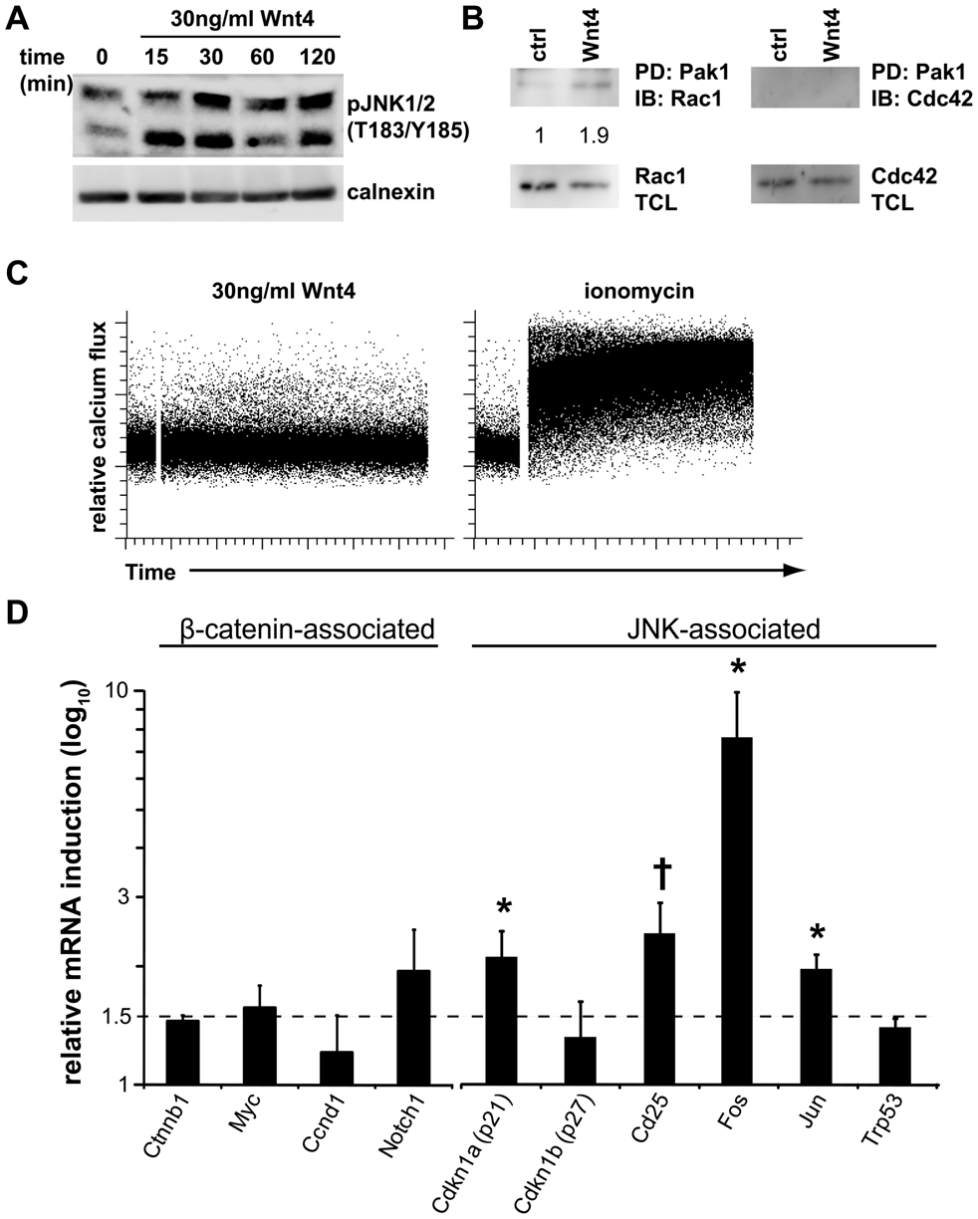
Wnt4 activates JNK but not calcium-dependent non-canonical signaling in hematopoietic progenitor cells. **A**) Representative western blot showing kinetics of JNK phosphorylation in the bone marrow HPC line EML after stimulation with 30 ng/ml recombinant Wnt4. Calnexin was used as a loading control. Similar results were obtained in at least three independent experiments. **B**) Representative western blot showing pulldown of GTP-linked forms of Rac1 (top left) and Cdc42 (top right) after stimulation with 30 ng/ml recombinant Wnt4 for 15 min. Total Rac1 and Cdc42 are shown at the bottom. The numbers correspond to the relative amount of Rac1-GTP (Rac1 in the pull-down/Rac1 in total cell lysate), with the unstimulated condition set as one. Similar results were obtained in three independent experiments. **C**) Representative flow cytometry data for intracellular calcium levels in EML cells after stimulation with 30 ng/ml recombinant Wnt4 or ionomycin as a positive control. Similar results were obtained in three independent experiments and with a range of different concentrations of Wnt4 (10–300 ng/ml). **D**) Expression of β-catenin-associated and JNK-associated transcripts in fetal liver LSKs in the presence of Wnt4 and 10 µM cycloheximide. Histogram represents the mean fold induction by Wnt4 from paired experiments (ratio Wnt4/control, standardized to HPRT expression; n = 5–6). The dashed line is set at 1.5. *P≤0.05; †*P*≤0.005.

Our previous results suggested that Wnt4 activated a non-canonical pathway. To confirm this was directly due to Wnt4, we stimulated EML cells, which are murine BM-derived multipotent progenitor cells [23], with recombinant Wnt4. Indeed, Jnk phosphorylation could be detected early on (Fig. 1A; peak at 30 min) in these cells. Jnk activation by Wnt proteins can occur via Rac1 as part of planar cell polarity (PCP) signaling, downstream of PKC in the calcium-dependent pathway, or downstream of the tyrosine kinase receptor Ror2 [1]–[3]. In a GTPase pulldown assay, we precipitated approximately twice as much active Rac1 from Wnt4-stimulated when compared to non-stimulated cells (Fig. 1B). In contrast, there was no Wnt4-specific activation of Cdc42 (Fig. 1B) or Wnt4-induced influx of Ca^2+^ (Fig. 1C), suggesting that Wnt4 might activate a PCP-like signaling pathway in hematopoietic progenitor cells.

It has been recently proposed that the nuclear localization of β-catenin may be dependent on Rac1 and Jnk2 [24]. To confirm that the activation of Rac1 and Jnk by Wnt4 did not result in the activation of β-catenin-dependent signaling in primary LSKs, we examined the induction of candidate target genes in the presence of the protein synthesis inhibitor cycloheximide in primary fetal liver LSKs by quantitative RT-PCR. Stimulation with Wnt4 did not result in direct activation of known canonical target genes, including cMyc, CyclinD1, or Notch1 (Fig. 1D). In contrast, four out of six genes (Cdkn1a^p21^, CD25, cFos, cJun) that have been shown to be regulated by Jnk activation downstream of other signaling pathways [25], [26] were induced by 2–8 fold (Fig. 1D). Together, the results from EML and primary LSKs demonstrate that Wnt4 activates a Rac1 and Jnk-dependent pathway that results in the activation of Jnk- but not β-catenin-dependent transcriptional program.

### JNK activity is necessary for Wnt4-mediated expansion of hematopoietic progenitor cells in culture

**Figure 2:**
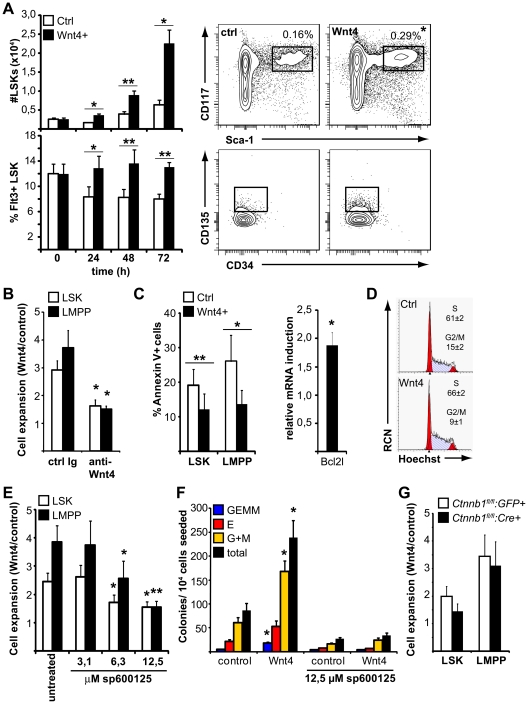
Wnt4 enhances LSK expansion in culture via JNK-dependent mechanisms. **A**) Fetal liver cells were cultured on NIH3T3 or NIH3T3-Wnt4 cells for 24–72 h and the proportion and number of LSKs were analyzed by flow cytometry at different time points. Histograms on the left show absolute numbers of LSKs and the percentage of LSKs expressing high levels of Flt3 (mean ± SEM; n = 7). Representative flow cytometry data for both Wnt4 (▪) and control (□) cultures after 48 h are shown on the right. Numbers in the upper-right corners of the FACS panels represent the percentage of LSKs over total live events. *P≤0.05, **P≤0.005, Wnt4 vs control (paired). **B**) The effect of 3 µg/ml anti-Wnt4 antibody or an isotype control on LSK (□) and LMPP (▪) expansion after 48 h of culture. *P≤0.05, antibody-treated vs control. **C**) Analysis of the impact of Wnt4 on LSK apoptosis in culture. Histogram on the left shows the percentage of AnnexinV positive LSK over live (PI-negative) cells from Wnt4 (▪) and control (□) cultures after 48 h (mean ± SEM; n = 5). Histogram on the right shows the fold induction of *Bcl2l* mRNA in fetal liver LSKs by a five-hour stimulation with Wnt4 (ratio Wnt4/control, standardized to HPRT expression; n = 4). *P≤0.05, **P≤0.005, Wnt4 vs control (paired). **D**) Cell cycle analysis of LSKs after 24 h of culture. Representative ModFit analyses of Hoechst staining from control (top) and Wnt4 cultures (bottom) are shown together with the mean ± SEM of the percentages of LSKs in S vs G2/M fractions of the cycle. None of the differences were statistically significant (n = 4). **E**) Fetal liver cells were cultured as above for 48 h in the presence of the specific JNK-inhibitor sp600125. Histogram shows the fold expansion of LSK (□) and LMPP (▪) in Wnt4 cultures relative to controls (mean ± SEM; n = 7). *P≤0.05, **P≤0.005, treated vs untreated. **F**) Fetal liver cells were cultured as above for 48 h; a fixed fraction of harvested cells were subsequently seeded in growth factor supplemented methylcellulose for CFC assays. Histograms show the number of different colonies [granulocyte/erythrocyte/monocyte/megakaryocyte (GEMM) (blue); erythroid (E) (red); granulocyte, monocyte or granulocyte/monocyte (G+M) (yellow)] and the total number of colonies (black) counted on day 7 (mean ± SEM; n = 3). *P≤0.05, Wnt4 vs other conditions. **G**) Analysis of fetal liver LSK and LMPP expansion in the presence (□) and absence (▪) of β-catenin. Histogram shows the fold expansion in Wnt4 cultures relative to controls (mean ± SEM; n = 4).

We previously reported that overexpression of Wnt4 led to *in vivo* expansion of LMPPs [16]. To determine whether the activation of Jnk was physiologically relevant in hematopoietic progenitor cells, we first wanted to establish an *in* vitro model where the effect of Wnt4 on LMPPs could be reproduced. To this end, we generated a stably transfected NIH-3T3-Wnt4 cell line. Wnt4 overexpression by NIH-3T3 cells did not alter their fibroblastic morphology or their expression of N-cadherin, E-cadherin, or β-catenin (Fig. S1). However, co-culture of fetal liver (FL) cells on NIH-3T3-Wnt4 resulted in increased Jnk phosphorylation when compared to cells that had been in contact with control NIH-3T3 cells (data not shown). We layered fetal liver cells on irradiated fibroblasts in the presence of the cytokine cocktail SCF/IL-3/IL-6 and analyzed the non-adherent CD45^+^ fraction at 24 h, 48 h and 72 h of co-culture for the number and percentage of LSKs. Wnt4 produced by stromal cells led to a time-dependent expansion of the LSK pool (Fig. 2A, top) and increased the proportion of Flt3^+^ LSKs (LMPPs) (Fig. 2A, bottom), similar to the phenotype we had previously observed in mice transplanted with *Wnt4*-transduced fetal liver cells [16]. The expansion was directly Wnt4-dependent as it could be blocked by an antibody able to recognize the native form of Wnt4 (Fig. 2B). Moreover, the presence of Wnt4 enhanced LSK survival (Fig. 2C) and resulted in the upregulation of the mRNA for the antiapoptotic protein Bcl-xL (*Bcl2l*), also reminiscent of the effect we reported before [16]; there was no significant difference in the percentage of LSKs in the S/G2/M phases of the cell cycle after 24 hours of co-culture (Fig. 2D), suggesting that Wnt4 did not directly induce LSK proliferation. Inhibition of Jnk activity in these cultures by increasing concentrations of the pharmacological inhibitor sp600125 largely abolished the effect of Wnt4 on primitive hematopoietic cells (Fig. 2E-F), thus demonstrating a functional relationship between Jnk activation and LSK expansion by Wnt4. The increase in LSK numbers in the presence of Wnt4-producing cells was further translated into a 3-fold increase in the number of colony-forming cells (CFCs), indicating that Wnt4 did not induce a disproportionate expression of Sca1 or cKit on cells that would have no HPC capacity (Fig. 2F). Conversely, deletion of β-catenin had no substantial impact on LSK expansion in response to Wnt4 (Fig. 2G). These results further confirm that Wnt4 influences LSK expansion and survival through a Jnk-dependent, β-catenin-independent pathway.

### Wnt4 and Jnk are both involved in BM LSK maintenance

**Figure 3:**
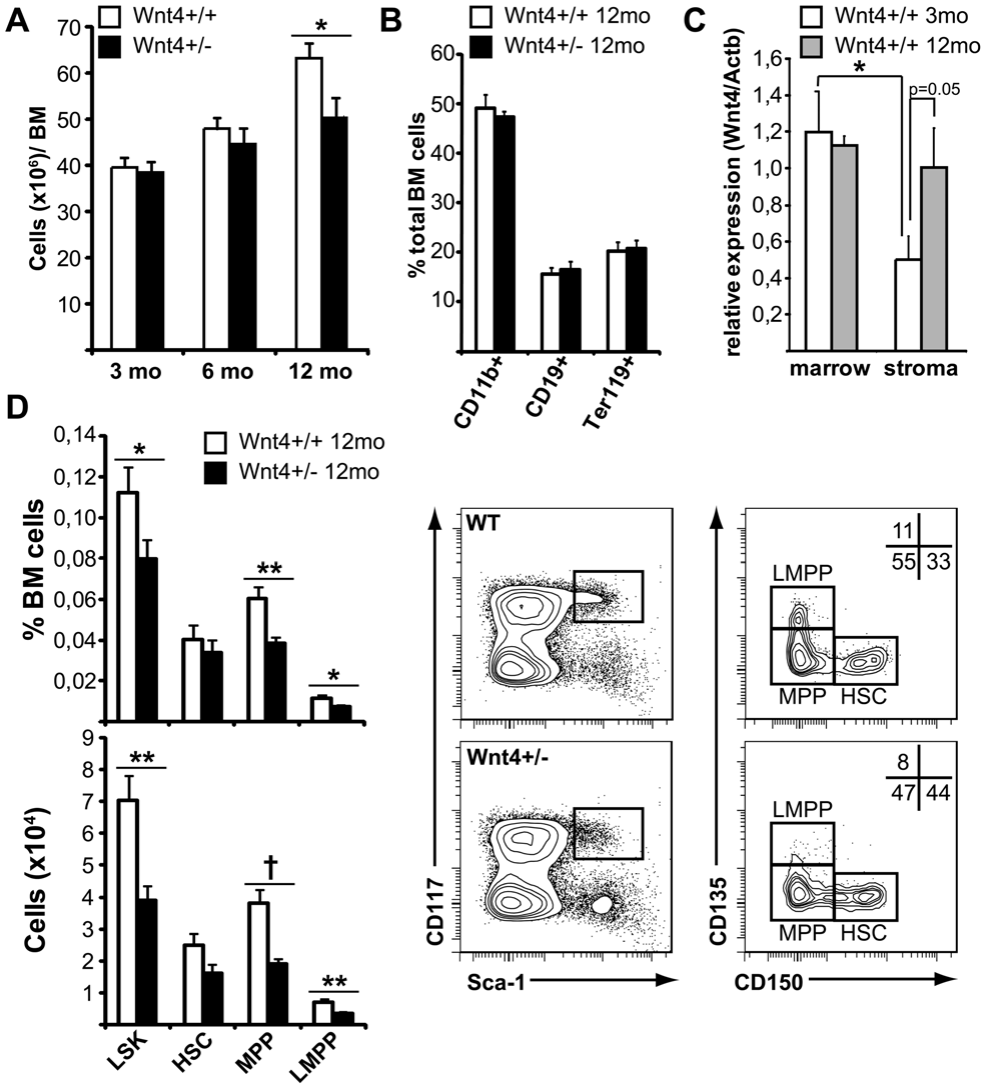
Wnt4 has an age-dependent effect on LSK maintenance in the BM. **A**) Total cell counts from BM (2 femora +2 tibiae) of adult *Wnt4^+/+^* (□) and *Wnt4^+/−^* (▪) mice of 3, 6, and 12 months of age. Histogram represents mean ± SEM from 6–7 animals per group. **B**) Analysis of the proportions of mature cells in the BM of 12-month-old *Wnt4^+/+^* (□) and *Wnt4^+/−^* (▪) mice. Histograms represent the percentage and number of CD11b^+^ myeloid cells, Ter119^+^ erythroid cells and CD19^+^ B lymphoid cells (mean ± SEM; n = 7). **C**) Analysis of Wnt4 expression by quantitative RT-PCR in the marrow and collagenase-digested bone (“stroma”) from adult *Wnt4^+/+^* mice of 3 (white) and 12 months of age (gray). Histogram represents mean ± SEM from four animals per group. **D**) Analysis of BM LSKs in 12-month-old *Wnt4^+/+^* (□) and *Wnt4^+/−^* (▪) mice. Histograms on the left show the percentage and number of total LSKs as well as HSC (CD150^+^CD135^−^), multipotent progenitors (MPP; CD150^−^CD135^−^) and LMPP (CD150^−^CD135^+^) subpopulations (mean ± SEM; n = 10). Representative flow cytometry data are shown on the right. Numbers within the flow cytometry panels represent the mean percentages of the different LSK subsets over total LSKs. *P≤0.05, **P≤0.005.

Our previous study had addressed the role of Wnt4 in fetal hematopoiesis and in emergency hematopoiesis after transplant [16]. Under both conditions, stem cells are actively expanding, in contrast to normal adult BM stem cells [27]. To evaluate whether Wnt4 has a non-redundant role in steady-state BM LSK biology, we assessed the effect of Wnt4 loss of function by comparing *Wnt4^+/−^* and *Wnt4^+/+^* adult mice (*Wnt4* ^−/−^ mice die within 24 h from birth*)*. Although young adult mice displayed no significant differences in their bone marrow (Fig. 3A and data not shown), a significant decrease in total BM cellularity was detected in older *Wnt4^+/−^* mice when compared to controls (Fig. 3A). This was not due to a problem with differentiation along any of the major BM lineages, as myeloid, erythroid and B lymphoid cells were all present at proportions that were comparable to those in normal littermates (Fig. 3B). Wnt4 expression was also stable in the *Wnt4^+/+^* marrow between three and 12 months. In contrast, collagenase-digested bone samples showed a two-fold increase in Wnt4 mRNA levels with age (Fig. 3C), suggesting that the late appearance of a phenotype could be due to age-related changes in the bone marrow microenvironment. Indeed, the percentage and number of BM LSKs (Fig. 3D), most notably the multipotent progenitors [MPPs; CD150^-^CD135(Flt3)^-^ LSKs] and LMPPs [CD150^-^CD135(Flt3)^hi^ LSKs], were decreased by approximately two-fold in 12-month-old *Wnt4^+/−^* mice when compared to age-matched *Wnt4^+/+^* mice. Thus, *Wnt4* haploinsufficiency led to a selective reduction in the size of the LSK pool in aged mice, possibly through decreased age-related expansion [28].

**Figure 4:**
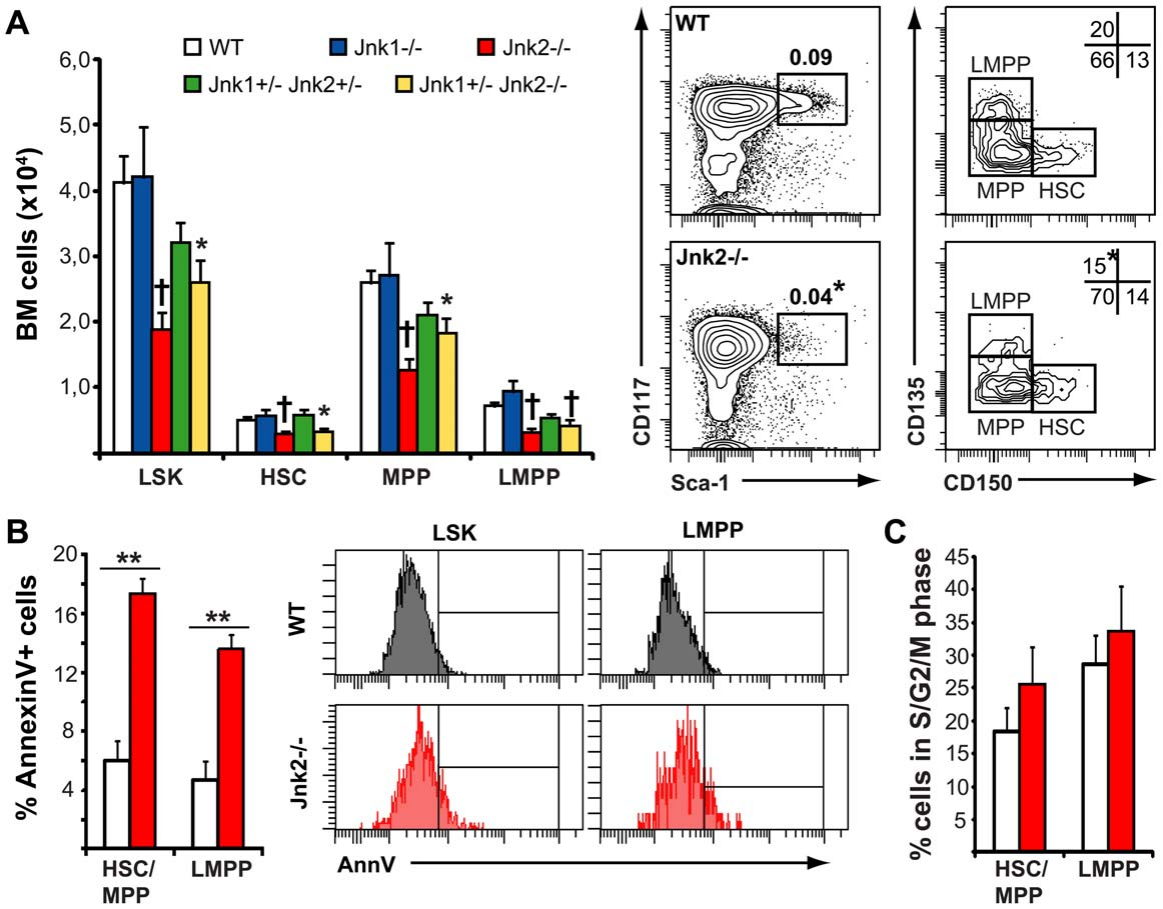
Jnk1 and Jnk2 have non-redundant roles in bone marrow LSKs. **A**) Flow cytometry analysis of BM LSKs in young adult (7–8-week-old) mice. Histograms on the left show the percentage and number of total LSKs as well as HSC (CD150^+^CD135^−^), MPP (CD150^−^CD135^−^) and LMPP (CD150^−^CD135^+^) subpopulations (mean ± SEM; n = 7–12). Representative flow cytometry data for *Jnk1^+/+^Jnk2^+/+^* (white bars) and *Jnk1^+/+^Jnk2^−/−^* mice (red bars) are shown on the right. Numbers within the flow cytometry panels represent the mean percentages of LSKs over total BM cells and those of the different LSK subsets over total LSKs. **B**) Analysis of LSK apoptosis in *Jnk1^+/+^Jnk2^+/+^* (white) and *Jnk1^+/+^Jnk2^−/−^* (red) mice. Histograms on the left show the percentage of AnnexinV positive LSKs and LMPPs (mean ± SEM; n = 5). Representative flow cytometry data are shown on the right. *P≤0.05, †*P≤*0.005. **C**) Cell cycle analysis of LSKs and LMPPs from *Jnk1^+/+^Jnk2^+/+^* (white) and *Jnk1^+/+^Jnk2^−/−^* (red) mice. Histogram represents the percentage of cells in the S/G2/M phases of the cell cycle (mean ± SEM; n = 5).

Earlier studies have identified JunB as a transcription factor in the Jnk pathway with the capacity to modulate progenitor cell differentiation and expansion [29]. However, the role of Jnk kinases in immature progenitor cells has not yet been established. We analyzed the BM from *Jnk1^−/−^, Jnk2^−/−^*, *Jnk1^+/−^ Jnk2^+/−^* and *Jnk1^+/−^ Jnk2^−/−^* mice (*Jnk1^−/−^ Jnk2^−/−^* mice are embryonic lethal [30]) and compared it to WT controls. The number of LSKs in *Jnk1^−/−^* and *Jnk1^+/−^ Jnk2^+/−^* BM were similar to WT mice, whereas *Jnk2^−/−^* and *Jnk1^+/−^ Jnk2^−/−^* BM presented marked decreases in both LSK percentages and numbers (Fig. 4A). In other cell types, Jnk2 deficiency has been shown to correlate with increased proliferation [31]. We did not detect consistent increases in the fraction of cycling *Jnk2^−/−^* LSKs (Fig. 4C); however, the percentage of apoptotic LSKs was significantly increased in *Jnk2^−/−^* BM (Fig. 4B). Together, our results indicate that endogenous Wnt4 and Jnk2 are involved in BM LSK homeostasis in adult mice, and that loss of Jnk2 diminishes LSK survival. In comparison, exogenous Wnt4 improves LMPP survival *in vitro* (Fig. 2B) and in hematopoietic chimeras [16].

### Wnt4 and Jnk improve hematopoietic reconstitution

**Figure 5:**
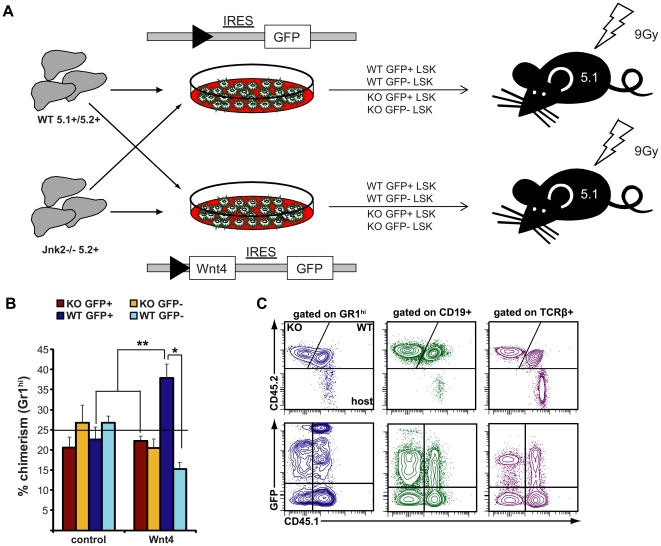
Wnt4 enhances long-term reconstitution via Jnk. **A**) Experimental design for competitive reconstitution. **B**) Presence of fetal liver LSK-derived progeny in the periphery 12 weeks post-transplant. The histogram represents the percentage of donor-derived CD11b^+^Gr1^hi^ granulocytes, separating *Jnk2^+/+^* (WT) and *Jnk2^−/−^* (KO) cells, transduced (GFP^+^) and non-transduced (GFP^−^) in control and Wnt4^+^ chimeras (mean ± SEM; n = 7 for Wnt4^+^ and n = 8 for control chimeras). The horizontal line is set at 25%, which was the relative input for each cell type [KO GFP+ (red); KO GFP- (yellow); WT GFP+ (dark blue); WT GFP- (light blue)]. *P≤0.05, **P≤0.005. **C**) Representative flow cytometry data from a Wnt4^+^ chimera, showing the presence of *Jnk2^+/+^* (CD45.1^+^ CD45.2^+^) and *Jnk2^−/−^* cells (CD45.1^−^ CD45.2^+^) in all lineages, persistence of host-derived T cells, and the broad distribution of GFP intensities in both *Jnk2^+/+^* and *Jnk2^−/−^* cells, suggesting oligoclonal distribution.

Although our results suggest that Wnt4 and Jnk2 have similar functions in BM LSK maintenance, we wanted to confirm a functional link between these two molecules *in vivo*. We thus compared the competitiveness of GFP- or Wnt4-transduced WT and *Jnk2^−/−^* LSKs in reconstituting an irradiated host (Fig. 5A). The transduced (GFP^+^) WT (CD45.1^+^CD45.2^+^) and *Jnk2^−/−^* (CD45.1^−^CD45.2^+^) fetal liver cells were transplanted in a 1∶1 WT:*Jnk2^−/−^* LSK ratio along with equivalent numbers of GFP^−^ LSKs (both WT and *Jnk2^−/−^*; thus each cell type represented 25% of the LSK input) into lethally irradiated CD45.1^+^CD45.2^−^ recipients (Fig. 5A). Multilineage reconstitution was determined in peripheral blood at 12 weeks post-transplant.

There was no significant difference in the total output of WT vs. *Jnk2^−/−^* LSKs and *Jnk2^−/−^* cells represented close to 50% of both granulocytes and B lymphocytes in control chimeras (Table 1). In these mice, GFP^-^ non-transduced cells had an advantage over GFP^+^ control-transduced cells, demonstrating that the retroviral vector itself did not provide a growth advantage. In comparison, GFP^+^ WT (CD45.1^+^ CD45.2^+^) cells were preferentially expanded in Wnt4^+^ chimeras (Table 1 and Fig. 5B, 5C) over GFP^+^ *Jnk2* ^−/−^ (CD45.1^−^ CD45.2^+^) cells and their GFP^−^ counterparts. Non-transduced GFP^−^ WT cells and *Jnk2^−/−^* cells produced granulocytes and B lymphocytes at similar frequencies; moreover, the overexpression of Wnt4 did not result in a growth advantage on a Jnk2-deficient background. Together, these results demonstrate a cell-autonomous Jnk2-dependent effect of Wnt4 in the myeloid and B lymphocytic lineages.

**Table 1:** Percentage reconstitution in peripheral blood of Wnt4^+^ and control chimeras 12 weeks post-transplantion.

Cell lineage	Transgene	WT transduced	WT non-transduced	*Jnk2^−/−^* transduced	*Jnk2^−/−^* non-transduced
Granulocytes	Control	22.6±3.2*	26.8±1.7*	20.6±2.7	26.7±4.5
	Wnt4	37.9±3.5*,†,‡	15.3±1.7*,‡	22.2±1.3†	20.5±2.2
B cells	Control	18.7±1.6*	33.5±2.2*	17.5±1.4	29.1±2.0
	Wnt4	32.2±3.2*,‡	22.9±2.3*,‡	21.3±2.7	23.0±2.3
T cells	Control	21.0±2.5†	40.8±3.5*,†	10.8±1.2†	15.0±2.0†
	Wnt4	28.9±2.6†	23.0±2.1*	15.0±2.3†	17.5±2.1
All lineages	Control	19.0±1.7*	32.8±2.2*	11.3±1.9	30.6±3.3
	Wnt4	31.0±2.0*,‡	19.8±1.7*,‡	23.1±3.5	19.1±2.5

See Figure 5 for experimental design. n = 7 for Wnt4+ and n = 8 for control group. Data are presented as mean ± SEM. A second, independent experiment produced similar results for myeloid reconstitution.

*P<0.01 Wnt4 compared to control (bilateral, unpaired *t* test);

† *P*<0.01 WT > KO (unilateral, paired *t* test);

‡ *P*<0.025 transduced (GFP^+^) > non-transduced (GFP^−^) (unilateral, paired *t* test).

The wide spectrum of GFP intensities observed by flow cytometry analysis (Fig. 5C) suggested that the recipient mice were not reconstituted by single transduced stem cells but were rather oligoclonal. To confirm these results, we analyzed the clonality and number of proviral integrations in sorted BM cells from both control and Wnt4^+^ chimeras, differentiating between WT and *Jnk2^-/−^* cells (Fig. S2). All samples analyzed consisted of at least 2–3 different clones, each of which harbored at least two proviral integrations. There were no major differences between controls and Wnt4-transduced cells; therefore, the effect of Wnt4 did not result from an increased viral transduction efficiency but rather from an improved repopulation capacity of the transduced clones (more progeny per stem cell). As a control, sorted GFP^-^ cells from the same mice had no detectable virus, confirming that they were indeed non-transduced.

In contrast to myeloid cells and B lymphocytes, Wnt4-transduced WT (CD45.1^+^ CD45.2^+^) T lymphocytes were not present at a significantly higher proportion than non-transduced WT T cells (Table 1 and Fig. 5C), consistent with our previous report [16]. The thymic cellularity of Wnt4^+^ chimeric mice was also increased when compared to control chimeras (data not shown). *Jnk2^−/−^* (CD45.1^−^ CD45.2^+^) T cells were under-represented in peripheral blood from both control and Wnt4^+^ chimeras, suggesting that Jnk2 is involved in T cell recovery after irradiation and transplant although it is not required for normal T cell development [32]. Thus, the role of Wnt4 in T cell development would appear non-cell-autonomous, in contrast to its effect on myeloid and B lymphocytic lineages, and may or may not directly involve Jnk2.

### Wnt4 signals can be transmitted through the planar cell polarity receptor Frizzled 6

In addition to the 10 different Frizzled receptor genes, unconventional receptors have also been reported to be activated by various Wnt family members. Of interest, Jnk phosphorylation has been reported both in the context of Frizzled and the Ror2 receptor tyrosine kinase [1]–[3]. No particular Frizzled receptor has been linked to Wnt4 in hematopoietic cells; moreover, no functional role has been reported for any single Frizzled in LSKs.

**Figure 6:**
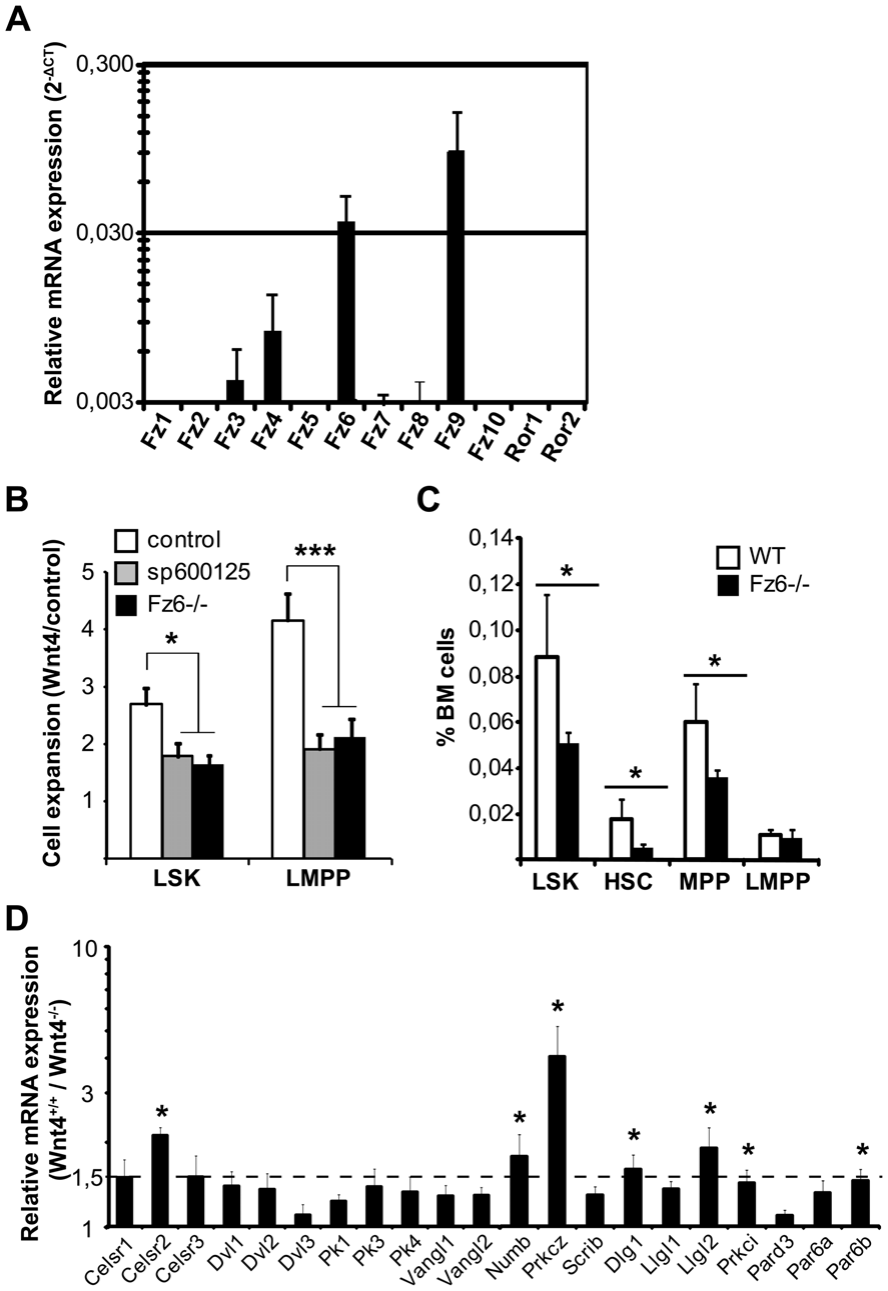
Wnt4 signals are mediated through a PCP receptor, Frizzled 6. **A**) Real-time RT-PCR analyses on sorted fetal liver LSKs, showing the expression levels of different Frizzled family members (mean ± SEM; n = 4). Data were normalized against HPRT expression. **B**) *Fz6^+/+^* (□) and *Fz6^−/−^* (▪) fetal liver cells were cultured for 48 h, as in Figure 2. Cells treated with the JNK inhibitor sp600125 (gray bars) are shown for comparison. Histogram shows the fold expansion in Wnt4 cultures relative to controls (mean ± SEM; n = 6). *P≤0.05, **P≤0.005, *Fz6^+/+^* vs. *Fz6^−/−^*. **C**) Analysis of BM LSKs in *Fz6^+/+^* (□) and *Fz6^−/−^* (▪) mice. Histogram shows the percentage of total LSKs as well as HSC (CD150^+^CD135^−^), MPP (CD150^−^CD135^−^) and LMPP (CD150^−^CD135^+^) subpopulations (mean ± SEM; n = 4). **D**) Expression of polarity-associated transcripts in *Wnt4^+/+^* and *Wnt4^−/−^* fetal liver LSKs. Histogram represents the mean fold increase in the presence of Wnt4 (ratio *Wnt4^+/+^*/*Wnt4^−/−^*; n = 4–6). The dashed line is set at 1.5. *P≤0.05.

We first examined the mRNA expression of the different Frizzled receptors as well as *Ror1* and *Ror2* in fetal liver LSKs. *Ror1* and *Ror2* were undetectable, two *Fz* were barely detectable (*Fz3* and *Fz4*) and two *Fz* were expressed at significant levels (*Fz6* and *Fz9*) (Fig. 6A). *Fz9*, the most strongly expressed Frizzled, has been linked to canonical Wnt signaling in B cell development [33]. *Fz6*, on the other hand, has been shown to control hair patterning in mice [34] and to negatively regulate β-catenin-dependent signaling, even in the context of Wnt molecules reputed to induce canonical signaling [35]. It has also been shown to bind Wnt4 [36].

Because *Fz6* appeared a likely candidate for transduction of non-canonical Wnt4 signals, we compared the *Fz6^−/−^* fetal liver LSKs to WT LSKs. We found that the Wnt4-mediated expansion of LSKs in culture was greatly reduced in the absence of Fz6, indicating that at least part of the Wnt4 signal in fetal liver LSKs was indeed transmitted through Fz6 (Fig. 6B). In fact, the effect of the lack of Fz6 was of the same magnitude as that of Jnk inhibition with 12.5 µM sp600125. Fz6 also appeared to be required for BM LSK maintenance as the percentage of LSKs was decreased by half in *Fz6^−/−^* animals when compared to WT mice (Fig. 6C). Interestingly though, the major LSK subpopulation affected was not LMPPs [in contrast to old *Wnt4^+/−^* mice (Fig. 3C)] but rather CD150^+^ LSKs, which predominantly contain quiescent long-term repopulating HSCs. This suggests that other receptors might also transmit Wnt4 signals, depending on the cell type.

Lastly we were interested in examining the expression of genes encoding PCP and polarity complex proteins in Wnt4-deficient LSKs by qRT-PCR. Out of twelve PCP genes, only *Pk2* was undetectable in fetal liver LSKs. The Flamingo-isoform *Celsr2* was downregulated by 2-fold in the absence of Wnt4 (Fig. 6D). Given that the activity of Dishevelled and Vangl can be regulated by post-translational modifications [37], the fact that their expression was not modified is not in itself a proof of them not being involved in Wnt4 signaling. More interestingly, we detected a differential expression of polarity complex genes *Numb*, *Prkcz*, and *Llgl2* and slight but statistically significant differences in the expression of *Dlg1*, *Prkci*, and *Par6b* (Fig. 6D). In all cases, the expression of polarity complex protein transcripts was decreased in the absence of Wnt4.

Together, these results identify Fz6 as a Wnt4 receptor in fetal liver LSKs and suggest that Wnt4 could be involved in the regulation of HPC polarity.

## Discussion

We report here that Wnt4 activated Rac1 and Jnk and directly induced a Jnk-associated but not β-catenin/TCF/LEF-associated transcriptional program in hematopoietic progenitor cells. We also demonstrate that Jnk activity and the presence of the receptor Fz6 were necessary for Wnt4-mediated LSK expansion, and that Wnt4, Jnk2 and Fz6 were all involved in BM LSK maintenance at steady state. In addition, Wnt4 improved the lineage-specific LSK competitive reconstitution capacity in a Jnk2-dependent manner. Our data provide a novel functional link between HPCs and Rac1/Jnk in the context of non-canonical Wnt signaling.

BM HSC can be found within specialized niches that provide them with signals associated with the maintenance of their “stemness”, self-renewal and integrity of their DNA. Although the precise nature of these signals and the proportion of HSC associated with either type of niche at any given time are still open for debate, they are generally accepted to come in two flavors: endosteal and vascular niches [38]–[40]. According to the prevalent dogma, quiescent HSCs would favor endosteal niches whereas more differentiated progenitors might be found in vascular niches [41]. In particular, increases in the numbers of osteoblasts have been associated with concomitant increases in the numbers of HSCs [38], [39], and osteoblasts produce factors that promote HSC quiescence and retain them in the niche [41]. A contrasting model has also been proposed, in which HSCs would be found in a relatively fluid equilibrium between endosteal and vascular niches at steady state and preferentially localize to the endosteal niche after injury due to irradiation [40], [42]. Two recent reports further specify that mesenchymal stem/progenitor cells compose an integral part of the hematopoietic stem cell niche [43], [44].

Of interest, both Wnt4 [45] and Jnk2 [46] have been shown to promote osteoblastogenesis and can thus be linked to osteogenic progenitors and to a putative stem/progenitor cell niche. However, the precise identity of the biologically relevant Wnt4-producing cell in the bone marrow is still unknown. Although the role of Wnt4 in promoting myeloid and B lymphoid development appears cell-autonomous (Table 1), and the leukocyte-rich marrow is the major Wnt4 producer in young mice (Fig. 3C), the effect of Wnt4 on LMPPs clearly has a non-cell-autonomous component (Fig. 2A). Indeed, we did not detect a linear correlation between the size of the LSK compartment or thymic cellularity and the percentage of Wnt4 expressing hematolymphoid cells in our previous study [16], although the experimental design did not allow for direct comparison of competitiveness between transduced and non-transduced cells. The potential influence of Wnt4 on osteoblastogenesis becomes thus particularly interesting in our HPC transfer model where the transplanted cells overexpress Wnt4. The re-establishment of the endosteal niche after total body irradiation has been shown to involve a transient expansion of osteoblasts [47]. Wnt4-producing HPCs could thus have a positive role in restoration of the HSC niches and improve hematopoietic recovery in a non-cell autonomous manner.

Fz6 has been linked to Wnt4 in a binding assay [36]; however, a functional relationship between the two had not been previously demonstrated. From a structural point of view, Fz6 belongs to the same family as *Drosophila* Fz, the receptor involved in the establishment of planar cell polarity rather than β-catenin/TCF signaling [1]. In mammalian cells, Fz6 is implicated in hair patterning in a manner replicating the PCP pathway in flies [34]. Furthermore, it has been shown to inhibit canonical Wnt signaling, even in the context of the canonical Wnt ligands and independently of β-catenin stabilization [35]. Wnt4 has also been shown to inhibit canonical Wnt signaling by targeting β-catenin to the membrane rather than nucleus [21]. Although the authors did not expand on the subjacent mechanisms, they suggested that Fz6 might be involved. Our data show that Fz6 was necessary for Wnt4-mediated LSK expansion *in vitro* and that the loss of Fz6 affected the size of the BM LSK pool, in particular the more primitive Flt3^−^ LSKs. Furthermore, although β-catenin was dispensable for Wnt4-mediated LMPP expansion *in vitro*, cell adhesion is central to the maintenance of HSCs in the niche; it is therefore conceivable that membrane β-catenin in HSC is necessary for the engraftment and the maintenance of cell-to-cell contact within the niche [9], [11], [48], while the transcriptionally active pool might drive expansion and cell cycle entry. Of note, Fz6 expression has been shown to be decreased on more differentiated progenitors [49], suggesting that Fz6 could transmit Wnt signals that favor sequestration of β-catenin to the membrane in HSCs while alternative receptors might be preferentially used in LMPPs which are rapidly cycling cells, presumably more loosely tied to the niche. Moreover, Fz6 was upregulated on BM LSKs from Wnt4^+^ chimeras [16], although we can only speculate on the consequences.

Although the expression of the majority of PCP genes was not altered in the absence of Wnt4, we identified seven polarity complex genes whose expression was downregulated in *Wnt4^−/−^* fetal liver LSKs. Of these, *Prkcz* has been identified as a positive regulator of HSC activity as its shRNA-mediated depletion resulted in impaired HSC repopulation and self-renewal [50]. On the other hand, the overexpression of *Numb* in thymocytes resulted in the loss of asymmetric cell division and increased thymic size [51]. Based on our previous results [16] and our results from the coculture experiments (Fig. 2), Wnt4 does not induce proliferation of progenitor cells but its effect appears strongest on proliferating cells. On one hand, the presence of Wnt4 improves the survival of the proliferating cells (Fig. 2C). On the other hand, the presence of Wnt4 might also modulate HPC polarity and thus division symmetry (Fig. 6D), although our results on the latter point are still preliminary. In both cases, the net result of Wnt4 signaling would be the expansion of the progenitor cell pool.

In conclusion, we provide a functional link between hematopoiesis and PCP-like Wnt signaling, and demonstrate that Wnt4 signals via Fz6, Rac1 and Jnk2 in immature hematopoietic cells, leading to increased LSK survival and improved competitive reconstitution. Based on our previous observations, the latter effect is likely to include both cell-autonomous and stroma-dependent components and we hypothesize that Wnt4 might be used post-irradiation to enhance not only the expansion of HPCs but also the restoration of BM niches.

## Materials and Methods

### Ethics Statement

All animal procedures were performed in accordance with the Canadian Council on Animal Care guidelines and were approved by the Comité de déontologie de l'expérimentation sur les animaux de l'Université de Montréal (protocols # 08-165 and 09-142). Generation of mouse NIH-3T3-Wnt4 cell line was approved by the Comité des Biorisques de l'Université de Montréal.

### Mice

C57BL/6 (B6; CD45.2^+^), B6.SJL-*Ptprc^a^Pep3^b^*/BoyJ (Ly5^a^) (B6.SJL; CD45.1^+^), B6.129S1-*Mapk8^tm1Flv^*/J (*Jnk1^−/−^*), B6.129S2-*Mapk9^tm1Flv^*/J (*Jnk2^−/−^*) and B6.129-*Ctnnb1^tm2Kem^*/KnwJ (*Ctnnb1^fl/fl^*; conditional β-catenin mutant) mice were purchased from The Jackson laboratory (Bar Harbor, ME). Wnt4 and Fz6 mutant mice were donated by Seppo Vainio at Oulu University and Jeremy Nathans at Johns Hopkins University School of Medicine, respectively. All mice were bred and housed under specific pathogen-free conditions in sterile ventilated racks at the Institute for Research in Immunology and Cancer.

### Cell culture and production of a Wnt4-producing cell line (NIH-3T3-Wnt4)

NIH-3T3 cells, kindly provided by Dr. G. Sauvageau [52], were transfected with the pUSE-Amp-Wnt4 plasmid (Upstate Biotechnology, Millipore, Billerica, MA) and selected for 10 days with 2.5 mg/ml Geneticin (Invitrogen, Burlington, ON). Expression of Wnt4 was confirmed by western blot and the strongest expressing clone was used as Wnt4-producing line (NIH-3T3-Wnt4) in further experiments. For co-culture experiments, 75–80% confluent monolayers in 6-well plate were irradiated at 15Gy and 3×10^6^ freshly extracted fetal liver cells were layered on top of the stromal cells in 2.5 ml DMEM supplemented with 15% FBS, 50 µg/ml gentamicin, 100 ng/ml SCF, 10 ng/ml IL-6, 6 ng/ml IL-3, and cultured for 24–72 h. For the CFC assays, FL cells were recovered from co-culture after 48 h, diluted 1/300 (the equivalent of 10^4^ cells on day 0) and plated in methylcellulose M3434 (Stem Cell Technologies, Vancouver, BC). Colonies were scored after 7 days according to size and morphology. EML cells were maintained in Iscove's Modified Dulbecco Medium (IMDM) supplemented with 20% horse serum, L-glutamine and BHK-KL cell conditioned medium as a source of stem cell factor. Both EML and BHK-KL cells were generously provided by David Williams at Harvard Medical School. Sp600125 was purchased from Calbiochem (EMD Chemicals, Gibbstown, NJ), recombinant Wnt4 and the anti-Wnt4 antibody (AF475) from R&D Systems (Cedarlane, Burlington, ON), cytokines from Peprotech (Cedarlane), and all other cell culture reagents from Invitrogen.

### β-catenin deletion

*Ctnnb1^fl/fl^* fetal liver cells were transduced with GFP control or Cre-GFP retroviruses as previously described [16]. The numbers of Lin^−^Sca1^+^cKit^hi^GFP^+^ cells were determined after 48 h infection and gene deletion was confirmed by semi-quantitative PCR on genomic DNA from sorted GFP^+^ cells using primers specific for the loxed allele or the deleted allele. The equivalent of 2.5×10^6^ total fetal liver cells were then layered on irradiated NIH-3T3 or NIH-3T3-Wnt4 cells (described above).

### Flow cytometry analysis and cell sorting

The antibodies were purchased from BD Pharmingen (San Diego, CA), Invitrogen, Cedarlane, eBioscience (San Diego, CA) or Biolegend (San Diego, CA). The following fluorochrome-conjugated antibodies were used to identify LSKs: PE-Cy7 and APC-Cy7 anti-CD117 (c-Kit, 2B8), Alexa700 anti-Sca1 (Ly6A/E; D7), PE anti-CD135 (Flt3, A2F10.1), Alexa Fluor 647 anti-CD34 (MEC14.7), and PE-Cy7 anti-CD150 (TC15-12F12.2). Biotin-labeled antibodies used in the lineage cocktail included**:** anti-CD8α (53-6.7), anti-NK1.1 (PK136), anti-TCRβ (H57), anti-CD11c (HL3), anti-TCRγδ (GL-3) and mouse lineage panel [CD3ε, CD11b, CD45R/B220, Ly6C/Ly6G (Gr-1), TER-119/erythroid cells (Ly-76)], except for fetal liver cells for which CD11b was omitted. The biotinylated lineage antibodies were detected with either PE-TexasRed, Alexa350, APC or APC-Cy7 conjugated streptavidin, depending on the combination of other fluorochromes used. For competitive reconstitution analysis, eFluor605 anti-CD45.1 (A20) and Alexa700 anti-CD45.2 (104) were used in combination with APC-Cy7 anti-Ly6C/G (Gr1; RB6-8C5), APC anti-TCRβ, PE anti-CD19 (1D3) and PE-Cy5 anti-CD11b (Mac1; M1/70) in the periphery, whereas APC-Cy7 anti-CD45.1 and PerCP-Cy5.5 anti-CD45.2 were used for BM LSKs. For cell cycle analysis, Hoechst 33342 was used following the manufacturer's instructions (Invitrogen). For apoptosis detection, surface labeled cells were washed with AnnexinV binding buffer and incubated with Alexa350 Annexin-V (Molecular probes). Propidium iodide was used to exclude dead cells from cell cycle and apoptosis assays. For calcium mobilization, cells were loaded with 10 µM indo-1 dye for 45′ at 37°C, washed and allowed to rest at room temperature. The baseline was recorded for 1′, followed by addition of recombinant Wnt4 or ionomycin as a positive control. Cells were analyzed on a three laser LSRII flow cytometer using DiVa software and sorted on a three laser FACSAria (BD Biosciences, Mountain View, CA).

### Competitive reconstitution experiments

Fetal liver cells were transduced with GFP control or Wnt4-IRES-GFP viruses as previously described [16]. The numbers of Lin^−^Sca1^+^cKit^hi^GFP^+^ cells were adjusted after 48 h infection to obtain equivalent numbers of wild-type (WT) and Jnk-deficient, GFP^−^ and GFP^+^ LSKs in the inoculum (25% input for each cell type). The equivalent of 2.5×10^6^ total fetal liver cells were then injected into the tail vein of lethally irradiated 8 to 12 week-old B6.SJL-*Ptprc^a^Pep3^b^*/BoyJ (Ly5.1) recipients. Mice were analyzed for donor chimerism at 12 weeks post-transplantation. Viral integration and copy number were determined by Southern blotting essentially as previously described [53]. In brief, genomic DNA extracted from sorted WT or Jnk-deficient GFP^+^ cells was digested with EcoRI, which cuts once in the provirus. DNA fragments were then detected with a GFP-specific radiolabeled probe. Sorted GFP^−^ cells from the same mice were used as a negative control.

### Quantitative RT-PCR

mRNA was prepared from fetal liver LSK cells (co-cultured for 5 h on NIH-3T3-Wnt4+ and control NIH-3T3 cells in the presence of cycloheximide for Fig. 1D or freshly isolated for Fig. 6A and 6D) sorted in Trizol reagent (Invitrogen) following the manufacturer's instructions. Total mRNA was reverse transcribed using the High Capacity cDNA Archive Kit with random primers (Applied Biosystems, Foster City, CA) as described by the manufacturer. A multiplex preamplification step was used to increase the quantity of specific cDNA target (TaqMan Pre-Amp Master mix Kit, Applied Biosystems) following manufacturer's instructions. Gene expression level was determined using the ABI PRISM® 7900HT Sequence Detection System (Applied Biosystems) together with primer and probe sets from Applied Biosystems and Universal ProbeLibrary. HPRT, GAPDH and TBP were used as endogenous controls (similar results were obtained with at least two of the three). The relative quantification of target genes was determined by using the ΔΔCT method [54].

### Western blots

EML cells were serum starved for 2 h and then either left unstimulated or stimulated with 30 ng/ml recombinant murine Wnt4 (R&D Systems). Cells were pelleted and lysed on ice in cold radio-immunoprecipitation buffer (50 mM Tris-HCl (pH 7.4), 1% Noniodet P-40, 0.25% sodium deoxycholate, 150 mM NaCl) supplemented with Complete protease inhibitor cocktail (Roche Molecular Biochemicals, Laval, QC, Canada) and phosphatase inhibitors (1 mM Na_3_VO_4_ and 5 mM NaF). The lysates were cleared by centrifugation and the protein content was measured by the Bradford method (Biorad, Missisauga, ON, Canada). Rac1 and Cdc42 pull-down was performed using the Rac1 Activation Assay Kit (Upstate Biotechnology) according to manufacturer's instructions. Samples were resolved by SDS-PAGE and immunoblotted with the indicated antibodies. Bands were revealed on LAS-3000 ECL Imager (Fujifilm) and analyzed using the Multi-Gauge software.

### Statistical analysis

Student's *t* test was used to determine statistical significance. *P* values of 0.05 or less were considered significant.

## Supporting Information

Figure S1 **Wnt4 does not affect the localization of β-catenin, E-cadherin and N-cadherin in NIH-3T3 cells. A**) Cells were fixed, stained with anti-β-catenin rabbit IgG antibody (shown in green) and with anti-E-cadherin mouse IgG antibody (shown in red), and acquired with a Zeiss LSM-510 confocal microscope. β-catenin expression localized to the cell-cell junctions, whereas E-cadherin localized to the perinuclear region in both NIH-3T3 and NIH-3T3-Wnt4 cells. **B**) Cells were fixed, stained with anti-β-catenin rabbit IgG antibody (shown in green) and with anti-N-cadherin mouse IgG antibody (shown in red), and acquired with a Zeiss LSM-510 confocal microscope. N-cadherin expression was found to colocalize with β-catenin at the cell-cell junctions. Images shown are representative of two to three experiments.
(TIF)

Figure S2 **The advantage provided by Wnt4 expression is not due to differences in transduction efficiency. A**) Schematic of the provirus and the strategy for proviral analysis. EcoRI cuts once in the provirus, upstream of GFP (which is used as probe to detect proviral integration). **B**) Southern blot of EcoRI-digested genomic DNA extracted from sorted WT transduced, KO transduced and non-transduced BM cells from representative chimeric mice. Each lane shows multiple bands of different intensities, representing multiple different proviral integrations. Lanes 1 and 2: GFP- non-transduced cells (negative control); Lanes 3–5: WT cells from control chimeras; Lanes 6–8: WT cells from Wnt4+ chimeras; Lanes 9 and 10: KO cells from control chimeras; Lanes 11 and 12: KO cells from Wnt4+ chimeras.
(TIF)
