# Supplementary figures and images for: Wnt4 Enhances Murine Hematopoietic Progenitor Cell Expansion Through a Planar Cell Polarity-Like Pathway
Source: PLoS One. 2011 Apr 26;6(4):e19279. doi: 10.1371/journal.pone.0019279 (PMC3082562; doi:10.1371/journal.pone.0019279)

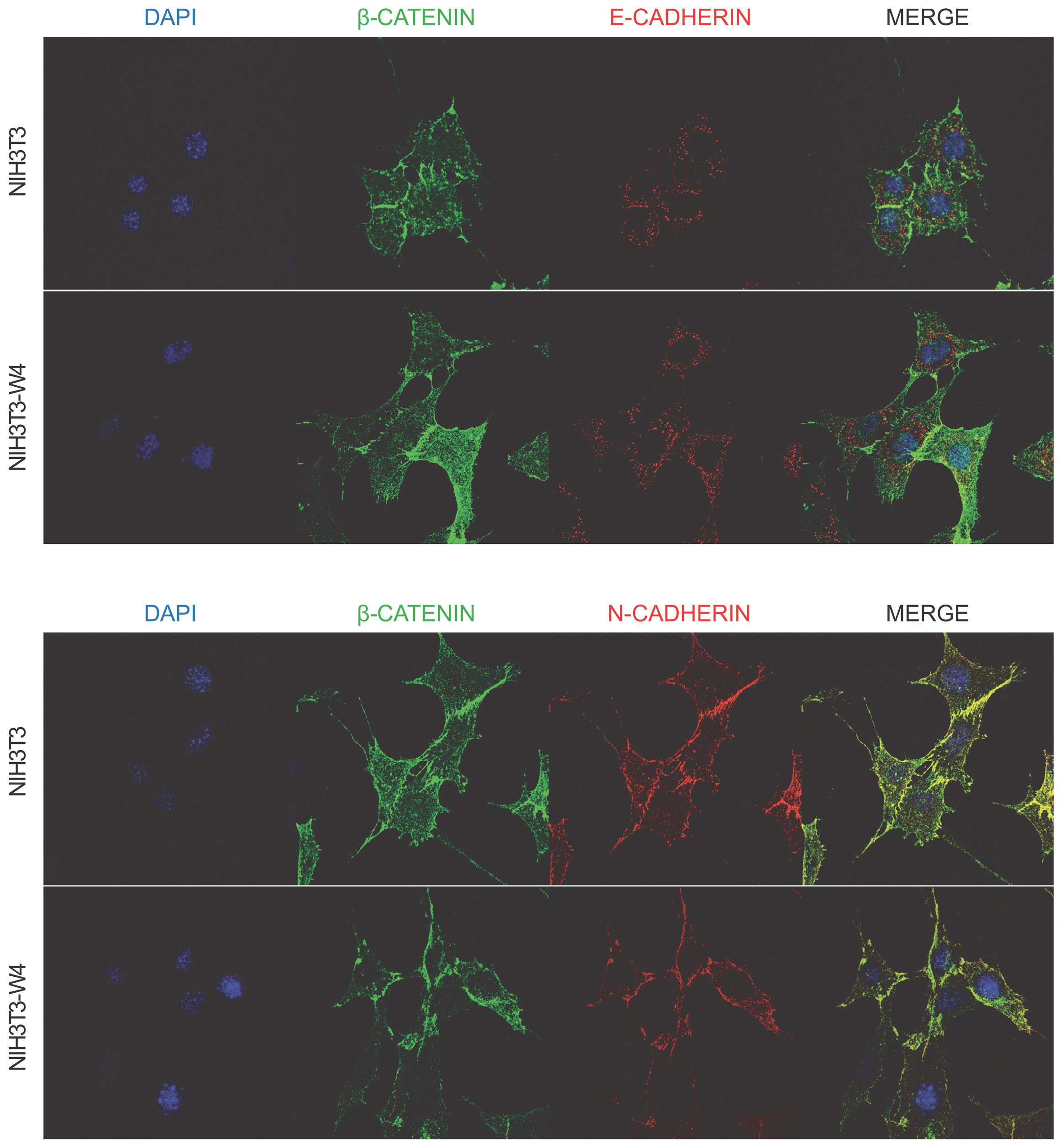

Supplement: Figure S1 — Wnt4 does not affect the localization of β-catenin, E-cadherin and N-cadherin in NIH-3T3 cells. A) Cells were fixed, stained with anti-β-catenin rabbit IgG antibody (shown in green) and with anti-E-cadherin mouse IgG antibody (shown in red), and acquired with a Zeiss LSM-510 confocal microscope. β-catenin expression localized to the cell-cell junctions, whereas E-cadherin localized to the perinuclear region in both NIH-3T3 and NIH-3T3-Wnt4 cells. B) Cells were fixed, stained with anti-β-catenin rabbit IgG antibody (shown in green) and with anti-N-cadherin mouse IgG antibody (shown in red), and acquired with a Zeiss LSM-510 confocal microscope. N-cadherin expression was found to colocalize with β-catenin at the cell-cell junctions. Images shown are representative of two to three experiments. (TIF) [file pone.0019279.s001.tif]

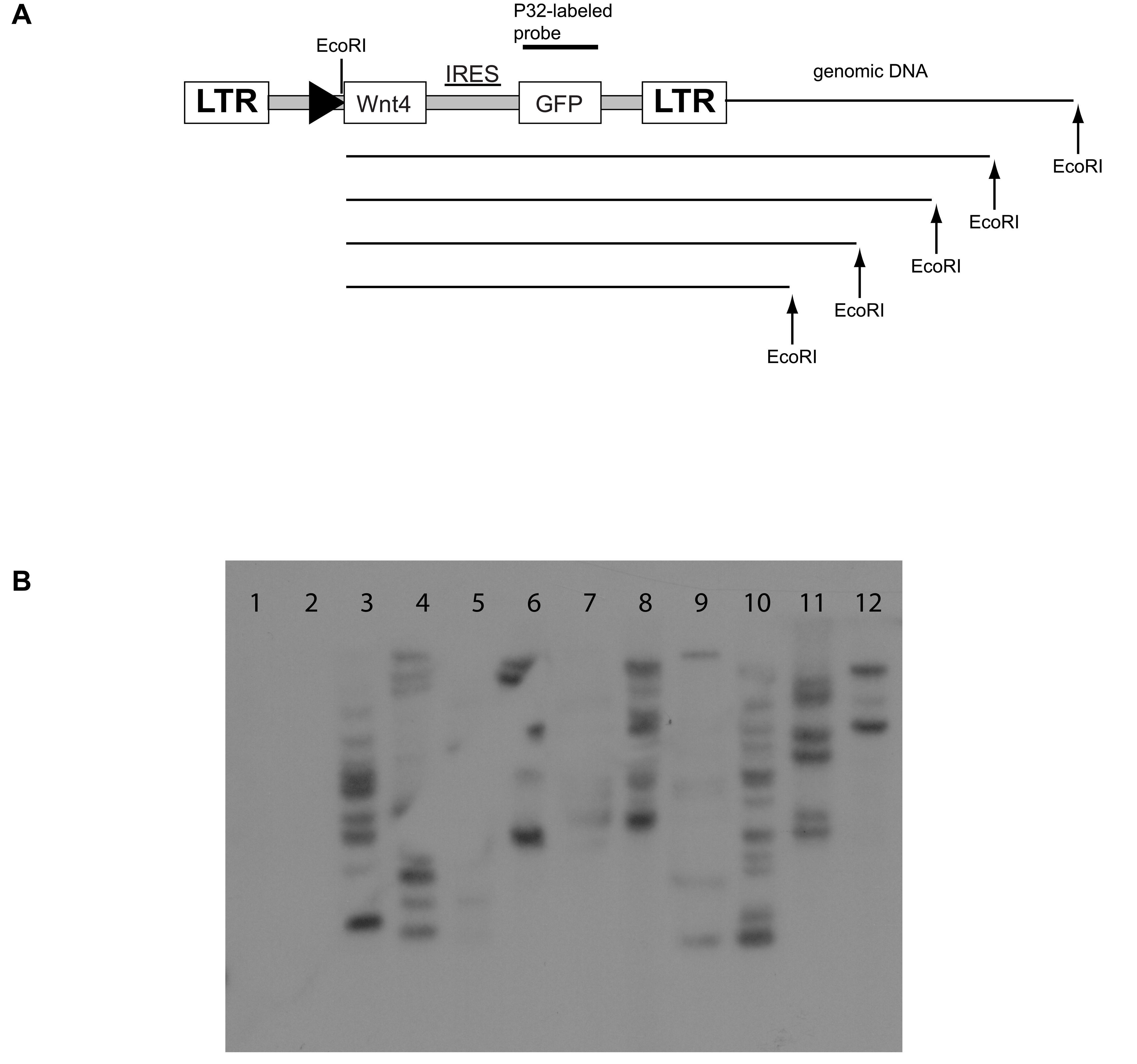

Supplement: Figure S2 — The advantage provided by Wnt4 expression is not due to differences in transduction efficiency. A) Schematic of the provirus and the strategy for proviral analysis. EcoRI cuts once in the provirus, upstream of GFP (which is used as probe to detect proviral integration). B) Southern blot of EcoRI-digested genomic DNA extracted from sorted WT transduced, KO transduced and non-transduced BM cells from representative chimeric mice. Each lane shows multiple bands of different intensities, representing multiple different proviral integrations. Lanes 1 and 2: GFP- non-transduced cells (negative control); Lanes 3–5: WT cells from control chimeras; Lanes 6–8: WT cells from Wnt4+ chimeras; Lanes 9 and 10: KO cells from control chimeras; Lanes 11 and 12: KO cells from Wnt4+ chimeras. (TIF) [file pone.0019279.s002.tif]
